# Comparison of the efficacy of soaking and drying methods for the disinfection of handwashing sinks in ICU

**DOI:** 10.3389/fpubh.2025.1602717

**Published:** 2025-08-01

**Authors:** Jieqiong Chen, Yu Chen, Xiaomin Xu, Zhaojun Xu, Chenxi Tong

**Affiliations:** ^1^Department of Intensive Care Unit, Ningbo No.2 Hospital, Ningbo, Zhejiang province, China; ^2^Infection Prevention and Control Department, Ningbo No.2 Hospital, Ningbo, Zhejiang province, China; ^3^Department of Nursing, Ningbo No.2 Hospital, Ningbo, Zhejiang province, China

**Keywords:** handwashing sinks, environmental surface contamination, intensive care unit, ATP, healthcare-associated infection, hospital environment, infection prevention and control

## Abstract

**Objective:**

This study aimed to investigate the effects of drying, soaking, and two combined disinfection methods on the disinfection efficiency of ICU handwashing sinks.

**Methods:**

From January to June 2024, eight handwashing sinks in the ICU were randomly divided into control group (*n* = 2), soaking group (*n* = 2), drying group (*n* = 2), and combination group (*n* = 2). The control group wiped with quaternary ammonium wipes only; the soaking group used chlorine disinfectant for 30 min based on the control group; the drying group was dried in a dryer for 15 min based on the control group; and the combined group was a combination of soaking and drying. Samples were collected once a month to compare the ATP fluorescence values and bacterial detection rates. Fisher’s exact test was used to compare categorical variables, Kruskal-Wallis’s test was used to compare continuous variables. Dunn’s post-test was used for pairwise comparison between groups.

**Results:**

The study found that before disinfection, no significant differences in ATP levels were observed among the four groups (*p* = 0.615). After disinfection, significant differences emerged (*p* < 0.001, *η*^2^ = 0.225), with the combined group showing the lowest ATP values and highest pass rate (100%). Dunn’s *post hoc* tests revealed the combined group had significantly lower ATP values compared to other groups (*p* < 0.01, Cohen’s *d* > 1.369). Bacterial detection showed an overall rate of 22.92% (11/48), with *Pseudomonas aeruginosa* being the predominant pathogen (81.82%, 9/11). The combined group had no bacterial isolates, while other groups had varying levels of contamination.

**Conclusion:**

Overall, all four methods in this study were found to be effective in disinfecting ICU handwashing sinks, but the combined method of soaking and drying was superior to the other three.

## 1 Introduction

The high prevalence of hospital-acquired infections (HAI) in the intensive care unit (ICU) has become a severe challenge, significantly elevating patients’ nosocomial mortality and morbidity and contributing to prolonged hospital stays and increased healthcare costs (1). Contamination of ward environments, particularly water-using areas such as handwashing sinks and showers, has become a recognized challenge in nosocomial infection prevention and control (2, 3). Hand hygiene is a crucial measure to reduce the spread of infections in healthcare settings (4), and almost all ICUs are equipped with handwashing sinks. Compared with the flowing water environment inside faucets and pipelines, the relatively stationary water environment on sink surfaces is more conducive to the adhesion of many microorganisms to form stable biofilms. Even after routine cleaning, the level of microorganisms remains high, making handwashing sinks a breeding ground for microorganisms and a potential source of infection in the long run (5, 6).

Therefore, it is crucial to thoroughly clean and disinfect the sink. Many studies have explored the effects of different disinfectants on the microbial colonization of hand sinks. According to research results and expert recommendations, chlorine-containing disinfectants and quaternary ammonium disinfectants are recommended for daily cleaning and disinfection (7), and a study found that combining two disinfectants is more effective (8). However, different disinfection methods seem to have varying effects on removing microorganisms and subsequent colonization of the sink’s surface (9). The conventional disinfection method usually uses wet wipes containing disinfectants to wipe the sink’s surface to achieve disinfection (10). Soaking in a disinfectant solution for more than 15 min is also a suitable method of disinfection in some studies (11). However, fewer studies have been conducted on drying sinks with dryers, and the results are still uncertain.

This study aims to compare the effectiveness of these methods in removing biofilms.

ATP testing is fast and efficient, providing results in 15 s for immediate monitoring and rapid response. Microbiological testing is highly accurate, directly identifying the type and number of microorganisms and providing detailed information on the source of contamination and microbiological testing have their strengths and necessities when cleaning and disinfecting surfaces, and combining the two provides a more comprehensive and accurate hygiene assessment to ensure the effectiveness of cleaning and disinfecting efforts. This study aims to compare the ATP fluorescence and microbial detection in ICU sinks after drying, soaking, and combined use of the two methods to investigate which disinfection method is better.

## 2 Methods

### 2.1 Study design

This study was a quasi-experimental study. From January to June 2024, eight handwashing sinks in the ICU were divided into four groups using a randomized numeric table method: control group (*n* = 2), soaking group (*n* = 2), drying group (*n* = 2), and combined group (*n* = 2). All ICU rooms are independently isolated single-bed units, and each handwashing sink is uniformly located at the entrance of these rooms. This standardized positioning ensures comparable levels of microbial load and usage frequency across sink groups, as they serve identical high-risk patient areas with equivalent water flow conditions. We collected ATP swabs from 8 sinks before and after disinfection every month to detect ATP fluorescence value, a total of 96 ATP swabs were collected for 6 months. In addition, cotton swabs were collected only after disinfection every month for microbial detection, a total of 48 cotton swabs were collected.

### 2.2 Intervention methods

#### 2.2.1 Control group

Initial cleaning was used a clean and disinfected towel to wipe the surface of the sink to remove water and debris. This was followed by disinfection using commercially available disposable wipes impregnated with a quaternary ammonium compound as the primary active ingredient (concentration: 2000–3,000 mg/L; wipe size: 20 cm × 22 cm). All accessible sink surfaces were thoroughly wiped to ensure complete disinfection coverage. This disinfection procedure was carried out twice daily, scheduled to avoid peak sink usage times. The procedures were executed by dedicated, professionally trained ICU environmental services staff. To minimize bias, staff members were blinded to the sink group assignments throughout the study period.

#### 2.2.2 Soaking group

Based on the control group, a 500 mg/liter concentration of chlorine disinfectant was completely soaked into the hand-washing sink, filled to the level of the overflow hole. Test paper was used to detect the concentration to ensure uniform distribution of the disinfectant. The soaking time was 30 min. Then, water was drained slowly for about 5 min. Finally, the disinfectant was removed from the surface with a disinfected wet towel.

#### 2.2.3 Drying group

A dryer was used in addition to the control group. The dryer had a temperature setting of 44°C and an action time of 15 min. During this process, a protective containment shield was installed to ensure thermal uniformity throughout the sink cavity. Real-time temperature monitoring confirmed that the temperature across all sink surfaces was maintained this level throughout the drying cycle.

#### 2.2.4 Combined group

The method of intervention for the combined group was to use the disinfection method of the control group first, then the soaking method and finally the drying method. The details are as previously described.

### 2.3 Sampling

#### 2.3.1 ATP sampling

ATP samples were collected immediately before and after each disinfection event of the handwashing sink. During sampling, a sterile 5 cm × 5 cm template was used to randomly select any 4 non-overlapped area on the sink surface. A single ATP swab was employed to vigorously swab the delineated area, moving horizontally and vertically across the surface five times each, while simultaneously rotating the swab head. After sampling, the ATP swab was returned to its tube. The tube was placed into the ATP detection instrument, and results were obtained on-site according to the manufacturer’s instructions.

#### 2.3.2 Microbiological sampling

Microbiological sampling was performed monthly for bacterial culture and antimicrobial susceptibility testing. Sampling times were scheduled to within 2 h after each disinfection. Similarly, using a sterile 5 cm × 5 cm template, 4 non-overlapped area was randomly selected on the sink surface. A cotton swab soaked with sterile physiological saline sampling solution was used to collect samples within this area. The cotton swab was inoculated onto Columbia blood agar plates. Using an inoculation loop, four areas were divided within the plate for pure separation. Immediately after the operation was completed, samples were sent to the microbiology laboratory for culture and testing.

Sample collection was performed by quality control personnel from the Hospital Infection Control Department. They did not participate in the intervention but had received relevant training. To avoid sampling bias, they were blinded to the group allocation.

### 2.4 Instruments

#### 2.4.1 ATP thresholds

According to the manufacturer’s instructions, ATP cleanliness was qualified as < 30 RLU after cleaning and < 100 RLU during use. The ATP pass rate was defined as the proportion of samples with post-intervention values < 30 RLU relative to the total samples tested. These thresholds align with literature indicating that instrument-specific benchmarks are critical for valid interpretation (12), though absolute values may vary across devices.

#### 2.4.2 Bacterial

Microbiology laboratory (with grouped information on their hidden hand washing sinks) testing with a VITEK-2Compact fully automated microbiology analyzer for isolation, cultivation, and identification of pathogenic bacteria. Growth intensity was semi-quantitatively assessed: 1 + (light growth): 10^3^ ~ 10^4^ CFU/mL, 2 + (moderate growth): 10^4^ ~ 10^5^ CFU/mL, 3 + (heavy growth): >10^5^ CFU/mL.

#### 2.4.3 Instruments source

Reagents Columbia blood plate (Zhengzhou Antu Bioengineering Co., Ltd.); VITEK-2Compact automatic microbiological analyzer (bioMérieux, France); ATP fluorescence detector (Hygiena system sure plus, USA); ATP sampling swabs (Hygiena Ultrasnap™).

### 2.5 Statistical analysis

All data in study were analyzed using SPSS 21.0. Continuous variables were expressed as means and standard deviation (SD), and the categorical data were expressed in percentages. Due to the small sample size, Fisher’s exact test was used to compare categorical variables, Kruskal-Wallis’s test was used to compare continuous variables. Dunn’s post-test was used for pairwise comparison between groups. According to Cohen (13) standards, for the effect size in Fisher’s exact test, we use Cramer’s V: 0.1 ≤ V < 0.3 indicates a small effect, 0.3 ≤ V < 0.5 indicates a medium effect, and V ≥ 0.5 indicates a large effect. For the Kruskal-Wallis non-parametric test, we used *η*^2^ for the effect size: *η*^2^ < 0.01 indicates a small effect, 0.01 ≤ *η*^2^ < 0.06 indicates a medium effect, and *η*^2^ ≥ 0.06 indicates a large effect. For Dunn’s *post hoc* multiple comparisons, the effect size Cohen’s d is used: *d* < 0.2 indicates a negligible effect; 0.2–0.5 indicates a small effect; 0.5–0.8 indicates a medium effect; and *d* ≥ 0.8 indicates a large effect. All statistics were performed using two sided tests, and the difference was considered statistically significant at *p* < 0.05.

### 2.6 Quality control

Fixed sterilization, collection, and testing staff were set up to reduce bias. Separating the first three from the researchers was realized to reduce subjective bias.

## 3 Results

### 3.1 ATP pass rate before and after disinfection of handwashing sinks in four groups

**Figure 1:**
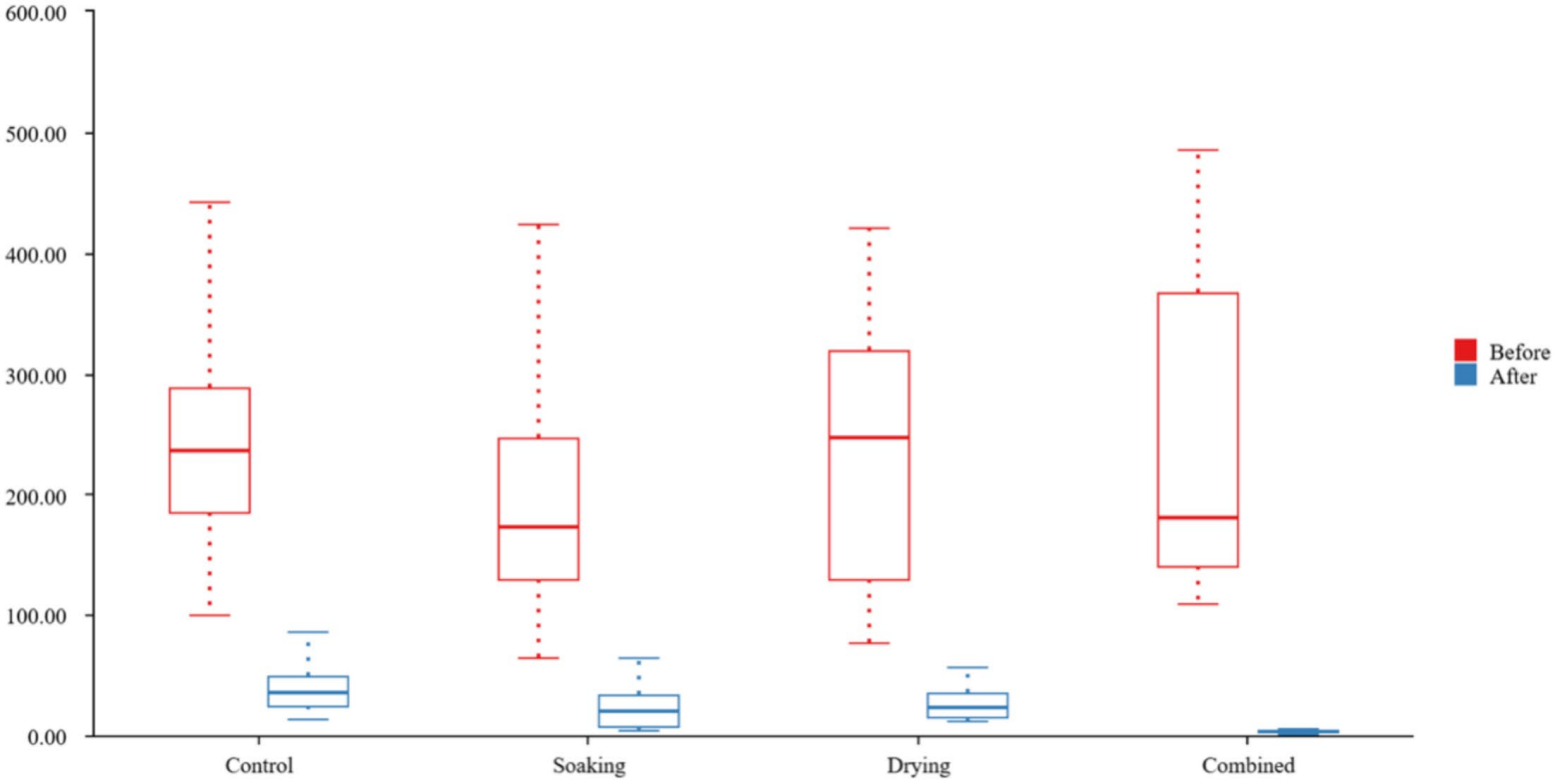
Boxplot of ATP fluorescence values before and after disinfection.

Before disinfection, four groups, two sinks per group, sampled six times each, resulting in 12 ATP swabs per group. The median ATP values were shown in Figure 1: Control group 235.5 (184.3, 287.5), Soaking group 172.0 (128.3, 246.0), Drying group 247.5 (128.8, 319.3), and Combination group 180.50 (140.0, 366.3). There was no statistically significant difference in ATP levels between the four groups before disinfection (*p* = 0.615).

After disinfection, 12 ATP swabs were collected in the same way. The median ATP values were shown in Figure 1: Control group 35.5 (23.5, 48.5), Soaking group 19.5 (7.5, 32.5), Drying group 22.5 (14.3, 34.8), and Combination group 2.5 (2.0, 4.0). The difference was statistically significant (*p* < 0.001), with an effect size *η*^2^ = 0.225. The significant effect size further indicates the statistical difference.

Pre-disinfection pass rates: soaking 16.67%, drying 8.33%, combination 0%, control 8.33%; no statistical difference. Post-disinfection pass rates: soaking 66.67%, drying 58.33%, combination 100%, control 33.33%; significant differences (*p* < 0.05). Details are in Table 1.

**Table 1:** Comparison of ATP fluorescence values and ATP pass rate before and after disinfection in four groups.

Group	Median ATP values	Pass rate	Median ATP values	Pass rate
	before disinfection	Before disinfection	After disinfection	After disinfection
Control (*n* = 12 + 12)	235.500	1 (8.333%)	35.500	4 (33.333%)
Soaking (*n* = 12 + 12)	172.000	2 (16.667%)	19.500	8 (66.667%)
Drying (*n* = 12 + 12)	247.500	1 (8.333%)	22.500	7 (58.333%)
Combine (*n* = 12 + 12)	180.500	0	2.500	12 (100%)
*H*	1.798		29.105	
*p*_1_/effect size	0.615/*η*^2^ = 0.038		<0.001*/*η*^2^ = 0.225	
*F*		2.182		11.932
*p*_2 /_ /effect size		0.536/Cramer’s V = 0.213		0.008*/Cramer’s V = 0.5

*N* = 12 + 12: It means that there are two sinks in each group, and six samples are collected before and after disinfection in every sink, that is, 12 samples are collected before and after disinfection in each group; H, Kruskal wallis’s test of ATP values of each group; *F*, Fisher’s exact test for ATP qualification rate of each group; *p*_1_, *p* value for Kruskal wallis’s test; *p*_2_, *p* value for Fisher’s exact test; effect size *η*^2^ < 0.01 indicates a small effect, 0.01 ≤ *η*^2^ < 0.06 indicates a medium effect, and *η*^2^ ≥ 0.06 indicates a large effect; effect size Cramer’s V: 0.1 ≤ V < 0.3 indicates a small effect, 0.3 ≤ V < 0.5 indicates a medium effect, and V ≥ 0.5 indicates a large effect. *p values < 0.05.

### 3.2 Comparison of ATP fluorescence detection values after disinfection in four groups

Testing revealed differences in ATP values among the four groups after disinfection. To further identify which group had lower ATP values, indicating better disinfection efficacy, we performed Dunn’s *post hoc* multiple comparisons and reported the effect size Cohen’s *d*.

As detailed in Table 2, the median difference between the Control group and the Combined group was 33 (*p* < 0.01; Cohen’s *d* = 2.412). This indicates that ATP values after disinfection were significantly higher in the Control group compared to the Combined group, with a statistically significant difference and a large effect size. Similarly, the median difference between the Soaking group and the Combined group was 17 (*p* < 0.01; Cohen’s *d* = 1.369), demonstrating significantly higher ATP values in the Soaking group versus the Combined group post-disinfection, with statistical significance and a large effect size. The median difference between the Drying group and the Combined group was 20 (*p* < 0.01; Cohen’s *d* = 1.544), indicating significantly higher ATP values in the Drying group compared to the Combined group after disinfection, with statistical significance and a large effect size.

**Table 2:** Pairwise comparison of ATP fluorescence values in the four groups after disinfection.

Group (1)	Group (2)	Median (1)	Median (2)	Difference (1–2)	*P_3_*	Cohen’s *d*
Control	Soaking	35.500	19.500	16.000	0.465	1.042
Control	Drying	35.500	22.500	13.000	1.000	0.868
Control	Combine	35.500	2.500	33.000	*p* < 0.01*	2.412
Soaking	Drying	19.500	22.500	−3.000	1.000	−0.175
Soaking	Combine	19.500	2.500	17.000	*p* < 0.01*	1.369
Drying	Combine	22.500	2.500	20.000	*p* < 0.01*	1.544

P_3_, *p* values for Dunn’s *t* test; effect size; Cohen’s *d*, *d* < 0.2 indicates a negligible effect; 0.2–0.5 indicates a small effect; 0.5–0.8 indicates a medium effect; and *d* ≥ 0.8 indicates a large effect. *p values < 0.05.

However, as shown in Table 2, pairwise comparisons among the Control, Soaking, and Drying groups revealed no statistically significant differences in ATP values.

### 3.3 Comparison of bacterial detection on handwashing sinks in four groups

A total of 48 swab samples were collected from the four groups. Detailed microbial detection results are shown in Table 3. In the control group (*n* = 12), 4 bacterial strains were isolated: 2 strains of *Pseudomonas aeruginosa* (both heavy growth: 3+), 1 strain of *Stenotrophomonas maltophilia* (heavy growth: 3+), 1 strain of *S. maltophilia* (moderate growth: 2+). In the soaking group (*n* = 12), 4 strains were identified: 2 strains of carbapenem-resistant; *P. aeruginosa* (CRPA, heavy growth: 3+); 1 strain of *P. aeruginosa* (heavy growth: 3+); 1 strain of *P. aeruginosa* (moderate growth: 2+). In the drying group (*n* = 12), 3 strains were detected: 1 strain of *P. aeruginosa* (heavy growth: 3+); 1 strain of *P. aeruginosa* (moderate growth: 2+); 1 strain of carbapenem-resistant *P. aeruginosa* (CRPA, heavy growth: 3+). No microorganisms were isolated from the combined group (*n* = 12).

**Table 3:** Detailed microbial detection results in four groups.

Group	Samples (*n*)	Strains isolated	Pathogen (growth intensity)	Resistance pattern
Control	12	4	*P. aeruginosa* (3+)	Susceptible to ceftazidime, ciprofloxacin, and meropenem
			*P. aeruginosa* (3+)	Susceptible to ceftazidime, ciprofloxacin, and meropenem
			*S. maltophilia* (3+)	TMP-SMX sensitive
			*S. maltophilia* (2+)	TMP-SMX sensitive
Soaking	12	4	*P. aeruginosa* (CRPA, 3+)	Carbapenem-resistant, resistant to meropenem, imipenem, and ceftazidime
			*P. aeruginosa* (CRPA, 3+)	Carbapenem-resistant, resistant to meropenem, imipenem, and ceftazidime
			*P. aeruginosa* (3+)	Susceptible to ceftazidime, ciprofloxacin, and meropenem
			*P. aeruginosa* (2+)	Susceptible to ceftazidime, ciprofloxacin, and meropenem
Drying	12	3	*P. aeruginosa* (3+)	Susceptible to ceftazidime, ciprofloxacin, and meropenem
			*P. aeruginosa* (2+)	Susceptible to ceftazidime, ciprofloxacin, and meropenem
			*P. aeruginosa* (CRPA, 3+)	Carbapenem-resistant, resistant to meropenem, imipenem, and ceftazidime
Combined	12	0	None detected	-

Growth intensity: 1 + (light growth): 10^3^ ~ 10^4^ CFU/mL, 2 + (moderate growth): 10^4^ ~ 10^5^ CFU/mL, 3 + (heavy growth): >10^5^ CFU/mL; CRPA, Carbapenem-resistant *Pseudomonas aeruginosa*; TMP-SMX, Trimethoprim-sulfamethoxazole.

The overall bacterial detection rate was 22.92% (11/48), with *P. aeruginosa* being the predominant pathogen (81.82%, 9/11). Among *P. aeruginosa* isolates: 33.3% (3/9) were carbapenem-resistant (CRPA). All CRPA strains showed heavy growth (3+), Heavy growth (3+) accounted for 77.78% (7/9) of *P. aeruginosa* isolates.

## 4 Discussion

This study compared four methods of cleaning and disinfecting ICU handwashing sinks and found that, except for the control group, the disinfection rate of the other three groups increased. After comparing the four methods two by two, it was found that the disinfection effect of the combined group was better than that of the other three.

As an efficient and highly sensitive detection technique, ATP fluorescence assay (14–16) has been widely used and validated in clinical practice to assess the degree of contamination of surfaces and sinks did not perform as expected, which differs from some existing studies. The reason for this may the effectiveness of cleaning and disinfection. In this study by ATP fluorescence assay, we observed that the handwashing sinks in the intervention group, the pass rate increased from 0–16.67% to 58.33–100.00%, and there was a significant difference among all three groups. However, there was no statistically significant difference in qualified rates before and after disinfection using quaternary ammonium wipes for wipe disinfection. In previous studies (17, 18), quaternary ammonium wipes demonstrated satisfactory results as a disinfection tool. However, this study on ICU handwashing be closely related to the unique moist environment of the sink. Previous studies (19) have focused on instrumentation or object surfaces in a dry state. In contrast, handwashing sinks provide favorable conditions for microbial survival and reproduction due to their frequent daily use and naturally wet state, which likely weakens the disinfection efficacy of quaternary ammonium wipes. In addition, the duration of disinfection may be one of the key factors influencing the effectiveness of disinfection, and sufficient contact time is essential for disinfectants to be fully effective in a given environment (20). In addition, we need to consider the potential impact of disinfectant resistance. As research progresses, more and more genomic evidence suggests that microorganisms exhibit innate and adaptive resistance to chemical disinfectants (21), and this resistance may further exacerbate the poor disinfection of quaternary ammonium wipes in humid environments. This study suggests that using quaternary ammonium wipes to wipe and disinfect ICU handwashing sinks briefly may not be sufficient to remove altogether or kill microorganisms adhering to moist surfaces, resulting in incomplete disinfection. A more comprehensive and effective cleaning and disinfection strategy is needed for handwashing sinks, which are a moist and highly frequented environment.

In this study, there was a statistical difference in comparing the cleaning and disinfection of ICU handwashing sinks before and after disinfection by the combined disinfection method. Comparative analysis by two-by-two comparison between the combined, control, soaking and drying groups reconfirmed the superiority of the combined group over any of the disinfection methods for cleaning and disinfection. In addition, the combined group showed a significant advantage in bacterial detection rate, with no microorganisms detected, which was statistically significant compared to the other three groups. This result strongly supports the effectiveness of co-disinfection methods in reducing microbial contamination in hospital environments. Combined use of different disinfectants may act together on microorganisms through multiple mechanisms, increasing bactericidal efficiency and reducing the development of resistance (22). In addition, the effect of hot bake drying in the combined method should not be overlooked, as the drying environment can further inhibit the growth of microorganisms (23). Contaminated environmental surfaces play an important role as vectors in the transmission of a wide range of healthcare pathogens, and effective cleaning and disinfection is one of the keys to reducing cross-infection.

In the present study, Paeruginosa had the highest detection rate in handwashing sinks, which coincides with previous studies (24, 25) and emphasizes the importance of P.aeruginosa as a common contaminant in hospital environments. Vitally, these sinks can act as reservoirs for pathogenic microorganisms, making thorough disinfection paramount. This risk is highlighted by the finding that 33.3% of these *P. aeruginosa* isolates were carbapenem-resistant strains (CRPA), all exhibiting heavy growth (3+), posing a severe challenge to clinical anti-infective therapy. Crucially, the results demonstrated that while bacteria were isolated from the control, soaking-only, and drying-only groups, the combined soaking-drying protocol achieved complete microbial eradication (0/12 samples), underscoring its exceptional efficacy against these virulent resistant pathogens. Therefore, it is strongly recommended to enhance surveillance for resistant bacteria in high-risk areas (e.g., ICUs) and implement this stringent combined disinfection protocol to prevent transmission. Fewer species of bacteria categories were detected in this study compared to other studies (26, 27), which may be related to the fact that only samples from ICU handwashing sinks were collected. Microbial contamination of handwashing sinks in different departments may vary due to differences in frequency of use, staff turnover, and patient type. In addition, the application of the four-zone delimitation method of pure species isolation and inoculation technique in this study, while helping to minimize the interference of mixed colonies, may also limit the diversity of strains detected to some extent. This method ensures that each colony is pure, facilitating subsequent identification and drug sensitivity testing. However, it may need to be combined with other sampling and analytical methods to assess environmental microbial contamination thoroughly.

Promoting soaking disinfection combined with hot drying strategies in handwashing sinks in ICUs and other high-risk areas of hospitals will not only help reduce the risk of bacterial contamination but also improve the safety of patients and healthcare workers. Additionally, it will help reduce the production and spread of drug-resistant bacteria. This approach necessitates dedicated equipment, such as thermally regulated dryers with containment systems, and consumes chemical disinfectants, incurring a modest cost compared to conventional wiping. However, these may be offset by reduced labor frequency and potential decreases in infection-related expenditures. In addition, it is essential to train medical logistics staff on how to properly clean and sanitize the sinks, how to accurately prepare chlorine-based disinfectants, and how to correctly operate the dryers. Such training is simple and cost-effective in the real ICU environment.

Despite detailed planning and implementation, some limitations must be considered. First, the small sample size was one of the main limitations of this study. Although monthly sampling over 6 months partially mitigated this issue, it could still lead to insufficient statistical power. To address this, we applied statistical methods to analyze the data, minimizing the impact of the limited sample size and ensuring the stability of the results. Future studies should adopt a repeated-measures crossover design, in which each sink undergoes different interventions in sequential time blocks. This approach would enable within-sink comparisons, reduce confounding factors, and enhance statistical power without requiring a larger number of sinks. Larger-scale studies would also be beneficial in confirming the effectiveness of the combined soaking and drying method and exploring its potential applications in different healthcare settings.

Second, there have been studies related to the contamination of clinical handwashing sink faucets, water supply pipes, and sink drains (5), but only handwashing sinks were chosen for this study. Additionally, although sinks are in similar areas of use and potential contamination areas, but the level of bacterial contamination in the environment around the sinks and the actual frequency of use of the sinks are not monitored, future studies should aim to comprehensively identify and control these potential confounding factors to improve the findings’ reliability.

## 5 Conclusion

In conclusion, the combined method of soaking and drying is associated with improving the cleaning and disinfection effect of the handwashing sinks in the ICU. However, the small sample size limits the generalizability of these results. Further larger randomized controlled trials—particularly using crossover designs—are needed to confirm these findings and optimize disinfection protocols.

## Data Availability

The raw data supporting the conclusions of this article will be made available by the authors, without undue reservation.
